# LncRNA Anxa10-203 enhances *Mc1r* mRNA stability to promote neuropathic pain by recruiting DHX30 in the trigeminal ganglion

**DOI:** 10.1186/s10194-024-01733-2

**Published:** 2024-03-04

**Authors:** YaJing Liu, Fei Liu, YiKe Li, YueLing Li, YuHeng Feng, JiaShuo Zhao, Cheng Zhou, ChunJie Li, JieFei Shen, YanYan Zhang

**Affiliations:** 1 State Key Laboratory of Oral Diseases, National Clinical Research Center for Oral Diseases, National Center for Stomatology, West China School of Stomatology, Sichuan University, Chengdu, 610041 China; 2 Department of Prosthodontics, West China Hospital of Stomatology, Sichuan University, Chengdu, 610041 China; 3 Laboratory of Anesthesia and Critical Care Medicine, Translational Neuroscience Center, West China Hospital, Sichuan University, Chengdu, 610041 China; 4 Department of Head and Neck Oncology, West China Hospital of Stomatology, Sichuan University, Chengdu, 610041 China

**Keywords:** Trigeminal ganglion, Long non-coding RNA, Neuropathic pain, RNA-binding proteins, DExH-box helicase 30, Melanocortin 1 receptor

## Abstract

**Background:**

Trigeminal nerve injury is one of the most serious complications in oral clinics, and the subsequent chronic orofacial pain is a consumptive disease. Increasing evidence demonstrates long non-coding RNAs (lncRNAs) play an important role in the pathological process of neuropathic pain. This study aims to explore the function and mechanism of LncRNA Anxa10-203 in the development of orofacial neuropathic pain.

**Methods:**

A mouse model of orofacial neuropathic pain was established by chronic constriction injury of the infraorbital nerve (CCI-ION). The Von Frey test was applied to evaluate hypersensitivity of mice. RT-qPCR and/or Western Blot were performed to analyze the expression of Anxa10-203, DHX30, and MC1R. Cellular localization of target genes was verified by immunofluorescence and RNA fluorescence in situ hybridization. RNA pull-down and RNA immunoprecipitation were used to detect the interaction between the target molecules. Electrophysiology was employed to assess the intrinsic excitability of TG neurons (TGNs) in vitro.

**Results:**

Anxa10-203 was upregulated in the TG of CCI-ION mice, and knockdown of Anxa10-203 relieved neuropathic pain. Structurally, Anxa10-203 was located in the cytoplasm of TGNs. Mechanistically, Mc1r expression was positively correlated with Anxa10-203 and was identified as the functional target of Anxa10-203. Besides, Anxa10-203 recruited RNA binding protein DHX30 and formed the Anxa10-203/DHX30 complex to enhance the stability of Mc1r mRNA, resulting in the upregulation of MC1R, which contributed to the enhancement of the intrinsic activity of TGNs in vitro and orofacial neuropathic pain in vivo.

**Conclusions:**

LncRNA Anxa10-203 in the TG played an important role in orofacial neuropathic pain and mediated mechanical allodynia in CCI-ION mice by binding with DHX30 to upregulate MC1R expression.

**Graphical Abstract:**

The up-regulated lncRNA Anxa10-203 in the trigeminal ganglion of CCI-ION mice interacts with DHX30 to contribute to the excitability of TG neurons and orofacial pain by enhancing *Mc1r* mRNA stability.

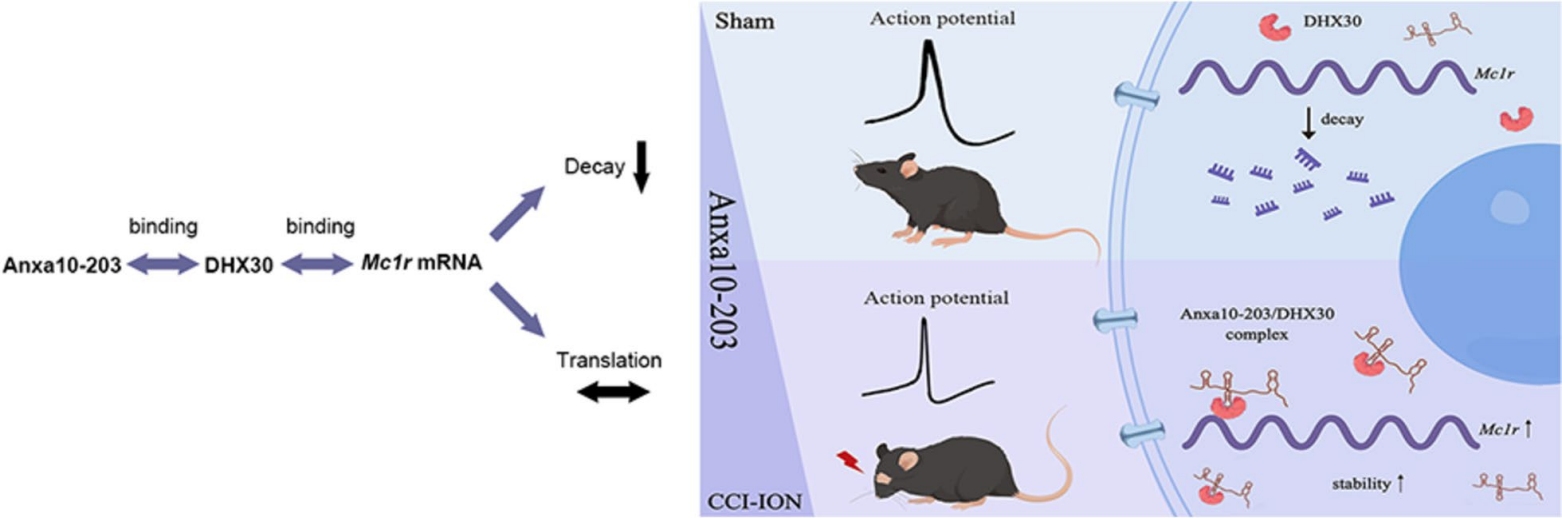

**Supplementary Information:**

The online version contains supplementary material available at 10.1186/s10194-024-01733-2.

## Background

Trigeminal nerve injury is one of the most serious complications in clinical orofacial operations, with an incidence of 0.5% to 37% [1]. About 50% of patients with trigeminal nerve injury have orofacial neuropathic pain (NP), which seriously affects their life quality [2, 3]. Due to the lack of effective intervention targets, clinical therapy is often unsatisfactory [4]. Therefore, it is of great importance to find new therapeutic targets and strategies for orofacial NP.

Long non-coding RNAs (lncRNAs) are involved in various physiological and pathological processes [5, 6]. Dysregulated expression of lncRNAs in the dorsal root ganglion (DRG), trigeminal ganglion (TG), and spinal dorsal horn has been detected in the preclinical models of NP [7, 8]. The imbalances of lncRNA transcription affect neuronal excitability and participate in the development of NP [9, 10]. Antisense oligodeoxynucleotides of lncRNAs have been reported to relieve nerve injury-induced hyperalgesia, indicating that lncRNAs are potential therapeutic targets for NP [11]. Our previous study have demonstrated the dysregulated IncRNAs in the TG of mice with orofacial neuropathic pain [12]. However, the functions of the above differentially expressed lncRNAs (DE lncRNAs) and their mechanism underlying orofacial NP remain to be further determined.

Post-transcriptional regulation mediated by RNA-binding proteins (RBPs) is an important regulatory pathway of lncRNAs [13]. DEAH box polypeptide 30 (DHX30) belongs to RNA helicases, which contribute to mRNA metabolism including ribosome biogenesis, global translation, and mitochondrial metabolism [14]. As an RBP, DHX30 was believed to participate in RNA-protein interaction [15]. The interaction between helicases and non-coding RNAs further expanded its biological functions [16, 17]. The regulation of mRNAs mediated by RBPs and the role of non-coding RNAs in this process is a relatively nascent field [18]. The interaction of DHX30 with non-coding RNA has not been reported yet, thus it is interesting how they contribute to NP.

Melanocortin receptor 1 (MC1R) is a G protein-coupled receptor originally found in melanocytes, which has been well-investigated in regulating pigmentation, UV responses, and melanoma risk [19]. Recently, the new function of MC1R in nociception was observed [20]. Mogil et al. [21, 22] reported the role of MC1R in pain for the first time, that humans and mice with loss of MC1R function were less sensitive to pain and had increased sensitivity to opioid analgesics. In humans, MC1R variants rs3212361 and rs885479 contributed to decreased orofacial pain sensitivity [23]. Besides, the intraventricular injections of α-MSH, which is the endogenous agonist of Mc1r, caused hyperalgesia, and injection of Mc1r antagonism also led to a high nociceptive threshold in wild-type mice similar to that of Mc1r^e/e^ mice [24, 25]. Therefore, MC1R may be a potential regulatory target in pain sensation. However, the underlying regulation mechanisms of MC1R remain limited.

In this study, the responsive expression of lncRNA Anxa10-203 in the TG neurons in mice with orofacial NP was reported. We focused on the RNA-protein interaction mechanism mediated by RBPs and further found that Anxa10-203 regulated the stability of Mc1r mRNA at the post-transcriptional level via recruitment of DHX30, which contributed to orofacial NP.

## Materials and methods

### Animals

The animal study was approved by the Ethics Committee of West China Hospital of Stomatology Sichuan University (Number: WCHSIRB-D-2022-021) and complies with the ARRIVE 2.0 guidelines. A total of 273 wild-type male C57BL/6 mice and 89 female C57BL/6 mice (6-8 weeks, 20-25g) were used for this study. Every single animal was defined as an experimental unit. All animals were kept in a specific pathogen-free experimental animal center (West China Hospital Experimental Animal Center) on a 12:12 light-dark cycle at 22 ± 1 °C. Food and water were available at liberty. In all assays, the animals were randomly allocated to the experimental groups using a random number table. The experimenters were blinded for the treatment groups during the analysis of the data in each assay.

### Cell culture and transfection

Bilateral TG was dissociated from connective tissue and minced. Then the tissue was enzymatically digested with 8 U/ml papain and incubated at 37°C for 20 minutes. An equal volume of complete medium was added to stop digestion and gently blow the digested tissue fluid several times with a sterile Pasteur pipette to mechanically dissociate neurons. Cell precipitation was obtained by centrifugation and resuspended in the neurobasal medium (Thermo Fischer, USA) containing 2% B27 (Invitrogen, USA) supplements and 1% GlutaMAX (Invitrogen, USA), and then plated in a twelve-well plate coated by poly-D-lysine (Invitrogen, USA) in 37℃ in humidified incubators with 5% CO2 and 95% air. The LV-Anxa10-203 (MOI = 5) and DHX30 siRNA or negative control siRNA (10 nM) were transfected into TG neurons (TGNs) on the second day. The Anxa10-203 oe+siDHX30 was the experimental group, and the Anxa10-203 oe+siNC (negative control siRNA) served as the control group. The LV-Anxa10-203 was purchased from Sangon (Shanghai, China). The siRNA and siNC were synthesized by GenePharma (Shanghai, China) and were transfected with HieffTrans *in vitro* siRNA/miRNA Transfection Reagent (Yeasen, China) following the manufacturer’s protocol. The sequences of siRNA are listed in Table 1.

**Table 1:** The sequences of siRNA/shRNA in this study

	Sense(5’-3’)
siAnxa10-203	GCCAAUUACUGUACUGAAUTT
siDHX30-1	GCUGGAAGGUGAUUCACGATT
siDHX30-2	CAAUGAGUACAGCGAGGAATT
siNC	UUCUCCGAACGUGUCACGUTT
AAV2/9-CMV-GFP-shDHX30	CAATGAGTACAGCGAGGAA
AAV2/9-CMV-GFP-shNC	CCTAAGGTTAAGTCGCCCTCG

### Chronic constriction injury of the infraorbital nerve (CCI-ION) surgery

Mice were anesthetized with isoflurane. For the CCI-ION group, two chronic gut ligatures (5-0) were placed loosely around the infraorbital nerve (ION) [26]. For the Sham group, the same surgical procedures were operated without ligation of the ION. The incision was sutured with 5-0 silk. The mice were placed on a heat recovery pad until they awoke from the anesthesia. Animals were monitored at least once daily after surgery.

### TG microinjection

The TG microinjection of mice was performed according to our previous study [27]. Briefly, after anesthesia, the siRNA Anxa10-203 (0.5 ug/μl), LV-Anxa10-203 (MOI = 5) or AAV2/9-CMV-GFP-shDHX30 (2E+9 vg/μl) were injected into the TG (coordinates: AP + 1 mm, ML + 4 mm, and DV -6.5 mm relative to bregma) under the guidance of a stereotaxic apparatus, and the maximum injection volume was 2 μl. A negative control siRNA or AAV2/9-CMV-GFP-shNC was used as control. The AAVs were synthesized by Xuanzun Bioscience (Chongqing, China). The siRNA was synthesized by GenePharma (Shanghai, China) and was transfected with Entranster^TM^-*in vivo* (Engreen Biosystem, China) following the manufacturer’s protocol.

### Behavioral tests

The Von Frey test was used to measure the 50% head withdrawal threshold (HWT) in response to mechanical stimuli according to our previous studies [27]. In brief, a series of Von Frey filaments were applied to the whisker pad area providing a force ranging from 0.04 g to 4 g. A positive response was recognized as sharp head withdrawal. The test was performed blinded.

### Reverse transcription and quantitative PCR

RNAiso regent (Takara, Japan) was used to obtain total RNA. cDNA was synthesized using PrimeScript™ RT reagent Kit (Takara, Japan). The RT-qPCR was performed by QuantStudio 7 Flex System (Applied Biosystems, USA). The primer sequences used are listed in Table 2. The relative expression of the target gene was normalized to GAPDH and calculated with the 2^-ΔΔCT^ method.

**Table 2:** The primer sequences used in this study

	Forward primer	Reverse primer
Anxa10-203	CCCACAACTTTGGCTGGGTA	TTCTGGGTGACATTTCCTCTTT
Mc1r	TCCTCGTGCCTCTATGGTGA	CTGGCCAAGGTTACGGATGT
DHX30	CTGGAGACTGTGTGGGTGTC	AGGTTCTCAAGAGGTGTGCG
GAPDH	AACAGCAACTCCCACTCTTC	CCTCTCTTGCTCAGTGTCCT
U6	GGAACGATACAGAGAAGATTAGC	TGGAACGCTTCACGAATTTGCG
Anxa11-204	TGGGAGGTTTAGTTGGTGTAAG	TGAAGAGCGTTGGAGATGATAG
Zbtb20-213	TGCCTGAACTTTGAAGCTGT	TGGGAAGACACCATCACCTCA
Tmem87a-204	TCGTTTCTTGGTGCAGGTCC	CTCTCCATCAACGACGGCAT
A630023A22Rik-202	AGAAACAAGCTCCTACATACACCA	TCCTGTCAAAGATCAGGCGG
Slc44a1-207	TCCAAACGAGAATGGAAGCCA	AAAGAACACATACCATCCCAATGC
Fez2-207	GCATTAGGGCTTCAGCAATGG	CCATGCCTCCTCGCATGAAT
Zbtb20-211	CCCGTCTCTGTGGTTTGTAAGG	TGACTGCCTGCCTCCCTAAT
TCONS_00110517	AGAGGTGTTCCAACATGCCA	AGGAAGGATGGTTTCGAGCA
TCONS_00090863	CAGAGGCTCTTTACTGGAACCT	AAGTTAGAGCTAATTGCAGCCC
Exosc10-206	TGCTGTAACCCAGTACCACC	ACACTGTACCTCGGCGCTA
TCONS_00081846	GGACCTTTCAGGCCAAGCTAA	CAACACTGGGATGTGTAGCTCT
Farp1-204	GAAGGTTCTGTTTGACGCCG	TGCTTCGGTCCACGATCTTC
Cars2-211	TCCACATTCTGCACAAGGGAC	CAGACCTGGAGTAACCTGCAA
TCONS_00261817	TGCAGAACACATTTGCCACAG	CCACTGCAGGATGCGATCT

### Subcellular cell fraction

The cytoplasmic and nuclear fractions of the TG were collected using a Nuclear and Cytoplasmic Extraction Reagents Kit (Beyotime, China) following the manufacturer’s protocol. RNA from cytoplasmic and nuclear fractions was extracted with RNAiso regent and analyzed by RT-qPCR. U6 and Gapdh were defined as nuclear and cytoplasmic control respectively.

### Immunofluorescence (IF) staining

The frozen sections were washed and permeabilized. The slices were blocked with 10% goat serum and then incubated with primary antibody overnight at 4℃. After washing with PBS, the sections were then incubated with a secondary antibody for 1 h. The images were captured with a laser scanning confocal microscope (LSCM, Olympus FV3000, Japan). The antibodies used are listed in Table 3.

**Table 3:** The antibodies used in this study

antibody	brand	dilution
anti-NeuN	Abcam, ab279296	1:1000
anti-MAP2	Abcam, ab254264	1:2000
anti-glutamine synthetase	Abcam, ab176562	1:1000
anti-CGRP	Cell signaling technology, 14959T	1:1000
IB4	Sigma, L214	1:100
Streptavidin-FITC	Sigma, S3762	1:100
anti-MC1R	Genetex, GTX108190	1:500
anti-DHX30	Abcam, ab85687	1:2000
anti-α-TUBB	Proteintech, 11224-1-AP	1:2000
peroxidase-conjugated secondary antibody	Signalway antibody, L3012	1: 5000

### RNA fluorescent *in situ* hybridization (RNA-FISH)

The fluorescence-conjugated probe for Anxa10-203 and *Mc1r* RNA was synthesized from GenePharma (Shanghai, China). After permeabilizing with 0.5% Triton, the tissue was incubated in a prehybridization solution then followed by a hybridization solution containing a 20 uM probe overnight at 37℃. the slides were finally washed with SSC solution. A LSCM (Olympus FV3000, Japan) was used to acquire the images.

### Western blot (WB) analysis

The samples were homogenized in a lysis buffer with protease inhibitors. The proteins were separated on 10% SDS/PAGE gel and then transferred to PVDF membranes. The membranes were blocked by 10% non-fat milk and incubated with the primary antibodies at 4℃ overnight, and further incubated with a secondary antibody. The antibodies used are listed in Table 3. Quantification of immunoblots was measured by ImageJ. The relative expression level of the target protein in all groups were normalized to α-Tubulin.

### RNA pull-down

The RNAs of the Anxa10-203, *Mc1r,* and four Anxa10-203 sequence segments were *in vitro* transcribed (Vazyme, China) and biotinylated (Thermo Scientific, USA). The Pierce magnetic RNA-protein pull-down kit (Thermo Scientific, USA) was used. Briefly, the biotinylated RNA was bonded to streptavidin magnetic beads (Thermo Scientific, USA). The TG lysates from CCI-ION mice were added into RNA-conjugated beads and incubated overnight at 4℃ with agitation. The RNA-interacting proteins were eluted and then analyzed by liquid chromatography-mass spectrometry (LCMS) and/or Western Blot. The input of the antisense RNA was defined as positive control and negative control separately.

### RNA immunoprecipitation (RIP)

Magna RIP Kit (Millipore, USA) was used to conduct the RNA-binding protein immunoprecipitation assay according to the manufacturer’s instructions. Briefly, the TG of CCI-ION mice was lysed by RIP lysis buffer. The magnetic beads conjugated with anti-DHX30 antibody were incubated with the tissue lysates on a rotator at 4℃ overnight. The magnetic beads conjugated with anti-IgG served as a negative control. The RNA was eluted from the beads, and the expression of Anxa10-203 and Mc1r was analyzed by RT-qPCR.

### Nascent protein synthesis assay

Click-iT Plus OPP Protein Synthesis Assay Kit (Invitrogen, USA) was applied. The Anxa10-203 oe+siDHX30 was the experimental group, and the Anxa10-203 oe+siNC served as the control group. The OPP working solution was added to the culture medium and incubated for 30 minutes. Then the cells were fixed and permeabilized. After washing, the OPP reaction cocktail was added and incubated for 30 minutes at room temperature. Images were acquired by LSCM (Olympus FV3000, Japan) and the relative fluorescence unit was assessed by a microplate reader (SpectraMax iD3, Molecular Devices, USA).

### Whole-cell patch-clamp recording

The electrophysiology was recorded by an Axopatch-700B amplifier and a Digidata 1440 digitizer (Molecular Devices, USA). The MC1R agonist BMS-470539 (BMS, 100 nM, MedChemExpress, China) was used, and the extracellular solution without BMS was defined as control [28]. The extracellular solution consisted of (in mM) NaCl 150, KCI 5, CaCl_2_ 2.5, MgCl_2_ 2, HEPES 10, and D-glucose 10 (pH = 7.4 by NaOH, 330 mOsm/L). The intracellular pipette solution contained (in mM) KCl 140, CaCl_2_ 1, MgCl_2_ 2.5, HEPES 10 and D-glucose 10, EGTA 11, Mg-ATP 5 (pH = 7.2 by NaOH, 310 mOsm/L). The resting membrane potential (RMP) was recorded for 3 min. The currents from -120 pA to 170 pA with an increment of 10 pA were injected into TGNs to evoke action potential (AP) [29]. The rheobase current was defined as the minimum current required to evoke the first AP. The AP threshold was obtained at d*V*/d*t*= 10 mV/ms. The action potential-related parameters including AP latency, the AP amplitude, AP half-width, afterhyperpolarization (AHP) time, and AHP amplitude are measured.

### Statistical analysis

Data were expressed as mean ± standard deviation. A two-tailed student’s t-test was conducted to compare the differences between the two groups. A repeated-measures ANOVA was used to detect the effect on HWT from both treatment effect and time effect, followed by one-way ANOVA for the difference between groups at each time point. Two-way ANOVA was applied to detect the expression level of genes from both treatment and time effect, followed by one-way ANOVA for differences between groups at each time point. And one-way ANOVA with the least significant difference (LSD) test was applied to analyze the differences among multiple groups. Statistical analyses were performed using GraphPad Prism software (v8.0.1, United States). *P* < 0.05 was considered as a statistical significance.

## Results

### The up-regulated expression of lncRNA Anxa10-203 in the TG was associated with orofacial pain induced by CCI-ION

**Fig. 1:**
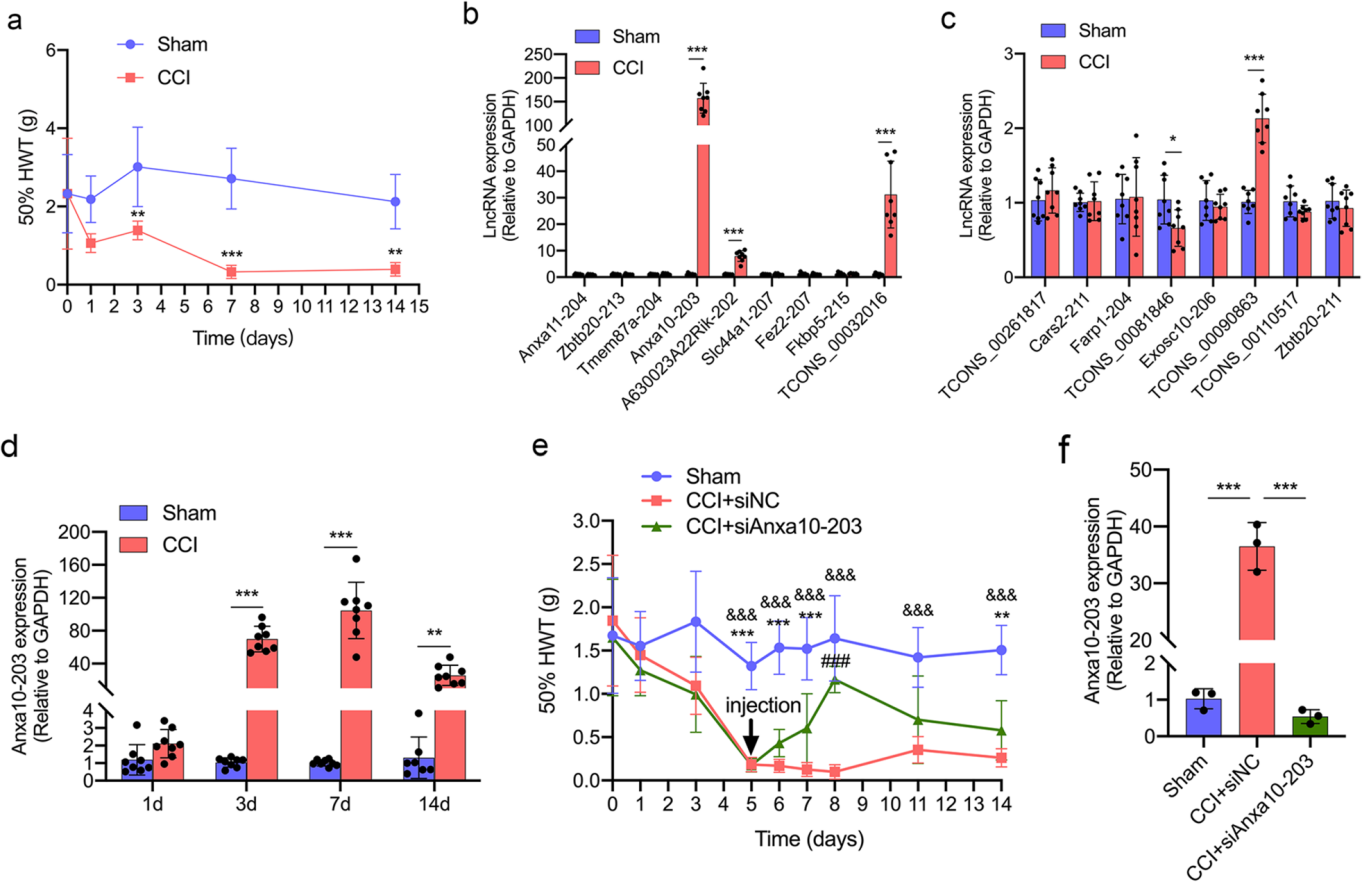
Anxa10-203 was up-regulated in the TG after CCI-ION. **a** Mechanical sensitivity threshold of ipsilateral whisker pad skin in Sham and CCI-ION mice. **P < 0.01, ***P < 0.001, *n* = 6/group. **b**, **c** The expression of the top 10 up-regulated (**b**) and 10 down-regulated (**c**) lncRNAs were verified in Sham and CCI-ION mice on day 7 after CCI-ION. *P < 0.05, ***P < 0.001, *n* = 7/group. **d** The expression of Anxa10-203 in the TG from mice of Sham and CCI-ION group at days 1, 3, 7, and 14. **P < 0.01, ***P < 0.001, *n* = 8/group. **e** The 50% WHT of the ipsilateral whisker pad region was increased in CCI-ION mice with Anxa10-203 silenced in the TG. *P < 0.05, ***P < 0.001, CCI+siAnxa10-203 versus Sham; ^&&&^*P* < 0.001, CCI+siNC versus Sham; ^###^*P* < 0.001, CCI+siAnxa10-203 versus CCI+siNC. *n* = 5/group. (f) The expression of Anxa10-203 decreased on day 4 after siAnxa10-203 injection. **P < 0.01, ***P < 0.001, *n* = 3/group

In our previous RNA-seq study [12], the top 10 up-regulated and 10 down-regulated DE lncRNAs were predicted (adjusted *P* value < 0.05, top 10 absolute log2-fold change in DE lncRNAs). To evaluate these DE lncRNAs in the TG of mice with orofacial pain, we first established the CCI-ION mice model. Von Frey test was applied to assess the mechanical pain threshold, and the results showed that CCI-ION induced significantly decreased 50% HWT on 3, 7, and 14 days (Fig. 1a). The expression of the top 10 up-regulated and 10 down-regulated DE lncRNAs was verified by RT-qPCR (data of Cdh4-204, Dleu2-212, Ninl-203, Gm28403-201 were abandoned for lack of specific primers), in which, TCONS_00081846 was significantly down-regulated, while Anxa10-203, A630023A22Rik-202, and TCONS_00032016 were significantly up-regulated (Fig. 1b-c). Considering the most striking fold changes of Anxa10-203, we selected Anxa10-203 for the following research. Then the expression of Anxa10-203 at different time points after CCI-ION was assessed. It was revealed that Anxa10-203 increased significantly on days 3, 7, and 14, and reached the peak on day 7 in the CCI-ION group (Fig. 1d). We further investigated whether TG microinjection of Anxa10-203 siRNA affected the development of CCI-ION-induced mechanical nociceptions. The behavioral tests demonstrated that the 50% HWT of mice increased after the siAnxa10-203 injection, and a significant difference was observed on the fourth day (Fig. 1e). The expression of Anxa10-203 in the TG of CCI-ION mice was significantly decreased four days after siAnxa10-203 injection (Fig. 1f). The data above indicated that CCI-ION induced increased Anxa10-203 expression in the TG, and Anxa10-203 knockdown relieved the pain induced by CCI-ION.

### lncRNA Anxa10-203 was expressed in the cytoplasm of neurons in the TG

**Fig. 2:**
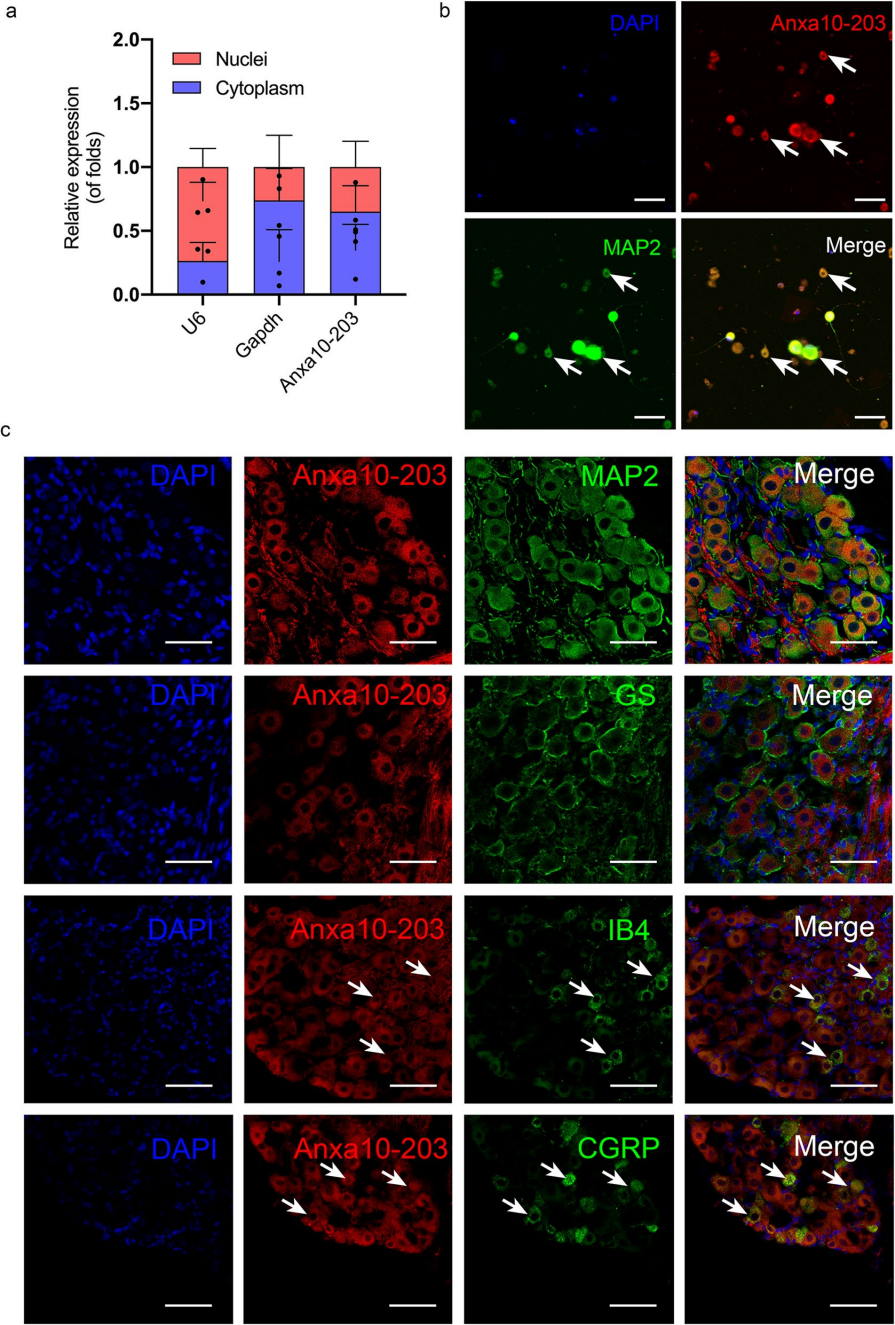
Anxa10-203 was expressed in the cytoplasm of TGNs. **a** The distribution of Anxa10-203 was assessed by the subcellular fraction assay. U6 and Gapdh were positive controls for the nucleus and cytoplasmic expression gene respectively. **b** FISH and IF staining demonstrated that Anxa10-203 was mainly expressed in the cytoplasm of TGNs *in vitro*. Scare bar = 50 μm. **c** Anxa10-203 RNA was co-located with MAP2, IB4, and CGRP *in vivo*. Representative immunopositive cells were identified by white arrows. Scare bar = 50 μm

The function of lncRNAs usually depends on their subcellular distribution, thus we further explored Anxa10-203 distribution in the TG [30]. To define the subcellular localization of Anxa10-203, the online algorithm LncLorator was used to predict first. The results showed that the score of cytoplasm is the highest, which is about 0.87 (Table 4). Consistent with the predicted results, the subcellular fraction assay further verified that Anxa10-203 was mainly expressed in the cytoplasm (Fig. 2a). Besides, RNA-FISH also showed that Anxa10-203 was mainly distributed in the cytoplasm of the cultured TGNs *in vitro* (Fig. 2b). *In vivo*, Anxa10-203 was co-located with MAP2 (a marker for neurons) but not with glutamine synthetase (GS, a specific marker for satellite glial cells) in the TG (Fig. 2c). Furthermore, The localizations of Anxa10-203 in non-peptidergic (labeled by the specific marker IB4) and peptidergic nociceptive neurons (labeled by the specific marker CGRP) were also observed (Fig. 2c). According to the data above, it was suggested that Anxa10-203 was mainly distributed in the cytoplasm of TGNs, which indicated that Anxa10-203 may play a role in post-transcriptional regulation.

**Table 4:** Prediction of subcellular localization of Anxa10-203 by LncLorator

Subcellular locations	scores
Cytoplasm	0.870730891308
Nucleus	0.0250258932124
Ribosome	0.0180279850876
Cytosol	0.0805810023881
Exosome	0.00563422800356

### Anxa10-203 RNA interacted with DHX30 protein in the TG

**Fig. 3:**
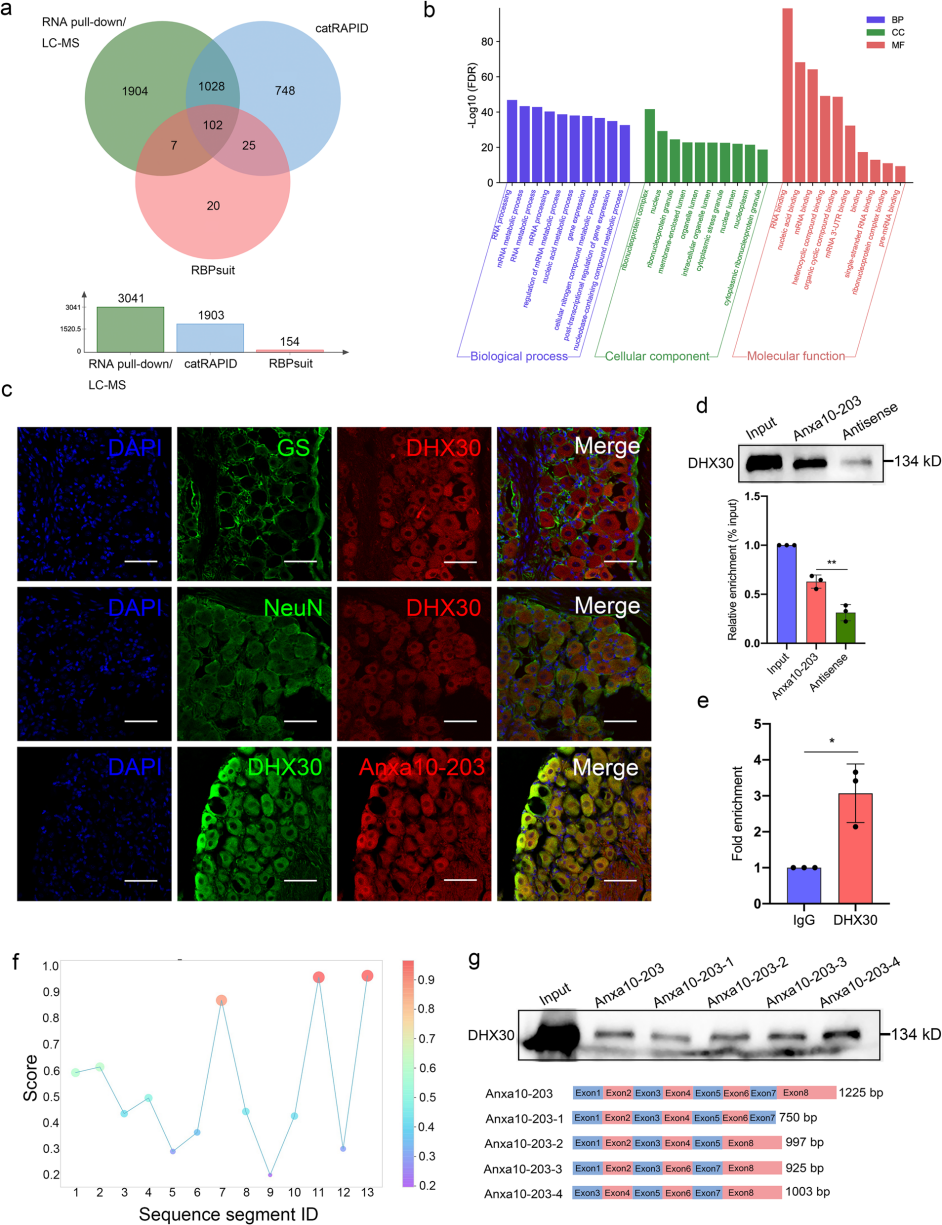
DHX30 protein interacted with Anxa10-203 RNA. **a** Venn diagram of the proteins from the RNA pull-down-LCMS and RNA-protein interaction online algorithm (CatRAPID and RBPsuite). **b** Gene Ontology analysis showed the top 10 enriched biological process, cellular component, and molecular function of these overlapped proteins. **c** RNA-FISH and IF staining revealed the co-localization of DHX30 protein and Anxa10-203 RNA in the cytoplasm of TGNs. Scare bar = 50 μm. **d** RNA pull-down assay assessed Anxa10-203 RNA interacted with DHX30 protein, the antisense served as control. **P < 0.01, *n* = 3/group. **e** RIP assay further confirmed that DHX30 protein interacted with Anxa10-203 RNA in the TG of CCI-ION mice. *P < 0.05, *n* = 3/group. **f** The scores of Anxa10-203-DHX30 binding sites predicted by RBPsuite. **g** The Anxa10-203 sequence segments that interacted with DHX30 protein were evaluated by RNA pull-down

LncRNAs-RBPs interaction is an important regulatory pathway of lncRNAs in post-transcriptional regulation, thus we focused on the LncRNA-protein interaction [13, 31]. To identify the binding proteins of Anxa10-203, RNA pull-down-LCMS and RNA-protein interaction online algorithms (CatRAPID and RBPsuite) were used. Totally, 3041, 1093, and 154 proteins were predicted by RNA pull-down/LC-MS, CatRAPID, and RBPsuite respectively, of which 102 proteins were overlapped (Fig. 3a). These overlapped proteins were notably enriched in RNA processing and RNA binding (Fig. 3b). Among the overlapped proteins, DHX30 with the highest score of catRAPID interaction propensity appeared to be the most likely binding protein of Anxa10-203. The co-localization of DHX30 protein and Anxa10-203 RNA in the cytoplasm of TGNs was demonstrated, indicating that there was spatial proximity (Fig. 3c). In the RNA pull-down assay, DHX30 protein was significantly pulled down by Anxa10-203 RNA compared with the antisense RNA (Fig. 3d). The interaction between Anxa10-203 and DHX30 in the TG of CCI-ION mice was further verified by RIP (Fig. 3e). RBPsuite was applied to predict the binding sites between DHX30 and Anxa10-203, and 5 binding sites were predicted and scored (Fig. 3f). Subsequently, serial sequence deletion of Anxa10-203 showed that the exon 8 of Anxa10-203 deletion decreased the binding of DHX30 protein (Fig. 3g), indicating that the exon 8 of Anxa10-203 was the main binding region of DHX30. The data shown above demonstrated the interaction between Anxa10-203 and DHX30 protein in the TG.

### DHX30 protein interacted with *Mc1r* mRNA

**Fig. 4:**
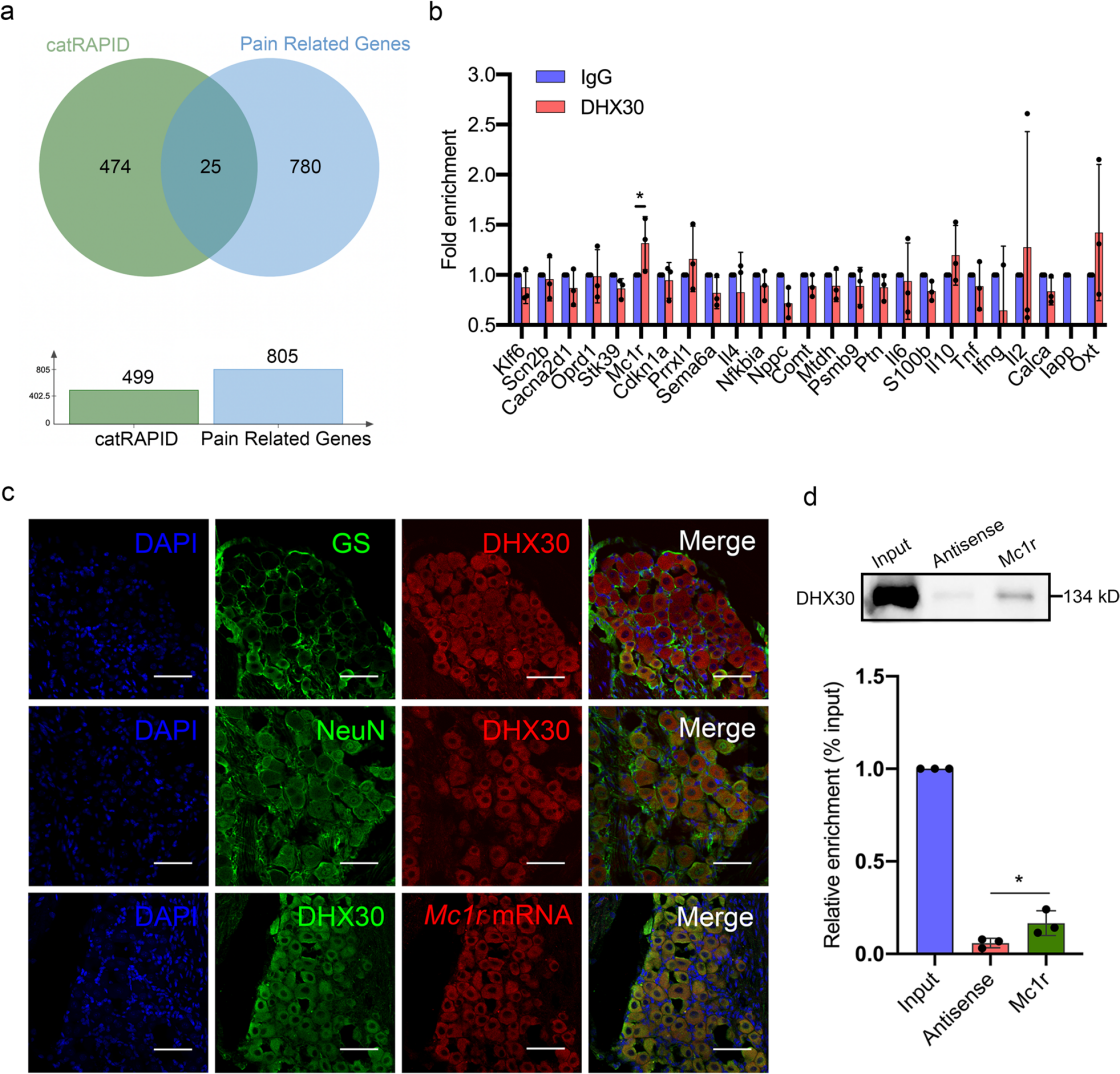
DHX30 protein interacted with *Mc1r* mRNA. **a** Venn diagram of the RNAs from the RNA-protein interaction online algorithm CatRAPID and pain-related genes. **b** RIP was used to validate the interaction between DHX30 and the overlapped RNAs. *P < 0.05, *n* = 3/group. **c** RNA-FISH and IF staining revealed the co-localization of DHX30 protein and *Mc1r* mRNA in the cytoplasm of TGNs. Scare bar = 50 μm. **d** RNA pull-down assay assessed *Mc1r* mRNA interacted with DHX30, the antisense served as control. *P < 0.05, *n* = 3/group

To predict the downstream mRNA of the Anxa10-203/DHX30 complex, CatRAPID was applied. 499 RNAs were predicted to interact with DHX30, in which 25 RNAs were overlapped in pain-related genes (Fig. 4a). RIP was used to verify the interaction between DHX30 and the overlapped RNAs, and the result showed that *Mc1r* mRNA was significantly enriched in DHX30-RNAs immunoprecipitations, suggesting the DHX30/Mc1r interaction in the TG (Fig. 4b). Further, FISH and IF assays verified that *Mc1r* mRNA and DHX30 protein were co-located in the cytoplasm of TGNs (Fig. 4c). Subsequent RNA pull-down demonstrated that *Mc1r* mRNA interacted with DHX30 as well (Fig. 4d).

### Up-regulated Anxa10-203 was associated with increased MC1R expression

**Fig. 5:**
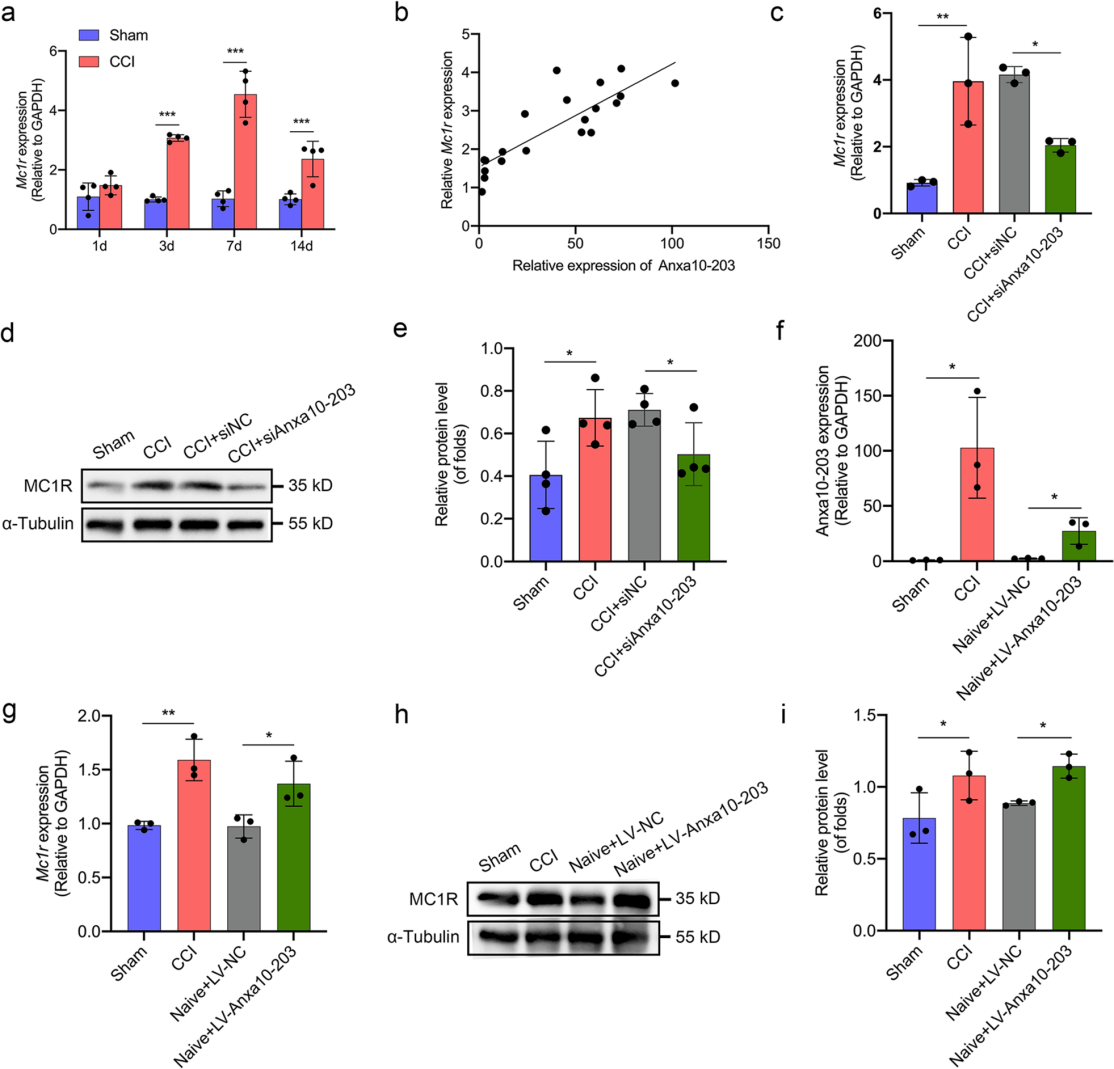
Anxa10-203 expression was associated with MC1R. **a** *Mc1r* expression in the TG on 1, 3, 7, and 14 days after CCI-ION. ***P < 0.001, *n* = 4/group. **b** Correlation analysis between Anxa10-203 and *Mc1r* in the TG by Pearson correlation analysis. *R*^*2*^ = 0.7023, *P <* 0.0001, n = 20. **c** Anxa10-203 knockdown reversed the *Mc1r* mRNA increase induced by CCI-ION. *P < 0.05, **P < 0.01, *n* = 4/group. **d**, **e** Anxa10-203 knockdown reversed the MC1R protein increase induced by CCI-ION. *P < 0.05, *n* = 4/group. **f** Anxa10-203 was increased 7 days after LV-Anxa10-203 microinjection in naive mice. *P < 0.05, *n* = 3/group. **g** *Mc1r* mRNA was significantly increased in TG induced by Anxa10-203 over-expression. *P < 0.05, **P < 0.01, *n* = 3/group. **h**-**i** Anxa10-203 over-expression induced increased MC1R protein in naive mice. *P < 0.05, *n* = 3/group

To demonstrate the expression pattern of MC1R, the expression of *Mc1r* mRNA at different time points was detected after CCI-ION. The results showed that *Mc1r* increased significantly at 3, 7, and 14 days after CCI-ION, and reached the peak on day 7 (Fig. 5a). Subsequently, Pearson correlation analysis verified that *Mc1r* mRNA was positively correlated with Anxa10-203 expression (*R*^*2*^ = 0.7023) and the result was statistically significant (*P <* 0.0001) (Fig. 5b). To further confirm the association between Anxa10-203 and MC1R, we knocked down Anxa10-203 and assessed MC1R expression. The results revealed the mRNA and protein of MC1R increased in the TG 7 days after CCI-ION (Fig. 5c-e). CCI-ION-induced increases of MC1R mRNA and protein were markedly reversed by siAnxa10-203 microinjection (Fig. 5c-e). We then explored whether the up-regulation of Anxa10-203 in the TG is sufficient to induce up-regulated MC1R. The significant increase of Anxa10-203 in the TG of naive mice was verified 7 days after LV-Anxa10-203 microinjection (Fig. 5f) and the expression levels of MC1R mRNA and protein in the TG were correspondingly increased (Fig. 5g-i). Besides, to explore whether the association between Anxa10-203 and MC1R was affected by sex difference, we also performed relevant studies on female mice. The RT-qPCR data in supplementary Fig. 1 ( see Additional file 1) indicated the positive correlation between Anxa10-203 and *Mc1r* mRNA in female mice, similar to that in male mice. To explore the effect of MC1R activation on neuron excitability, we also performed electrophysiology in supplementary Fig. 2 (see Additional file 1), which indicated that MC1R activation promoted the intrinsic excitability of TGNs, resulting in the development of orofacial pain.

### DHX30 enhanced *Mc1r* mRNA stability in the condition of Anxa10-203 over-expression

**Fig. 6:**
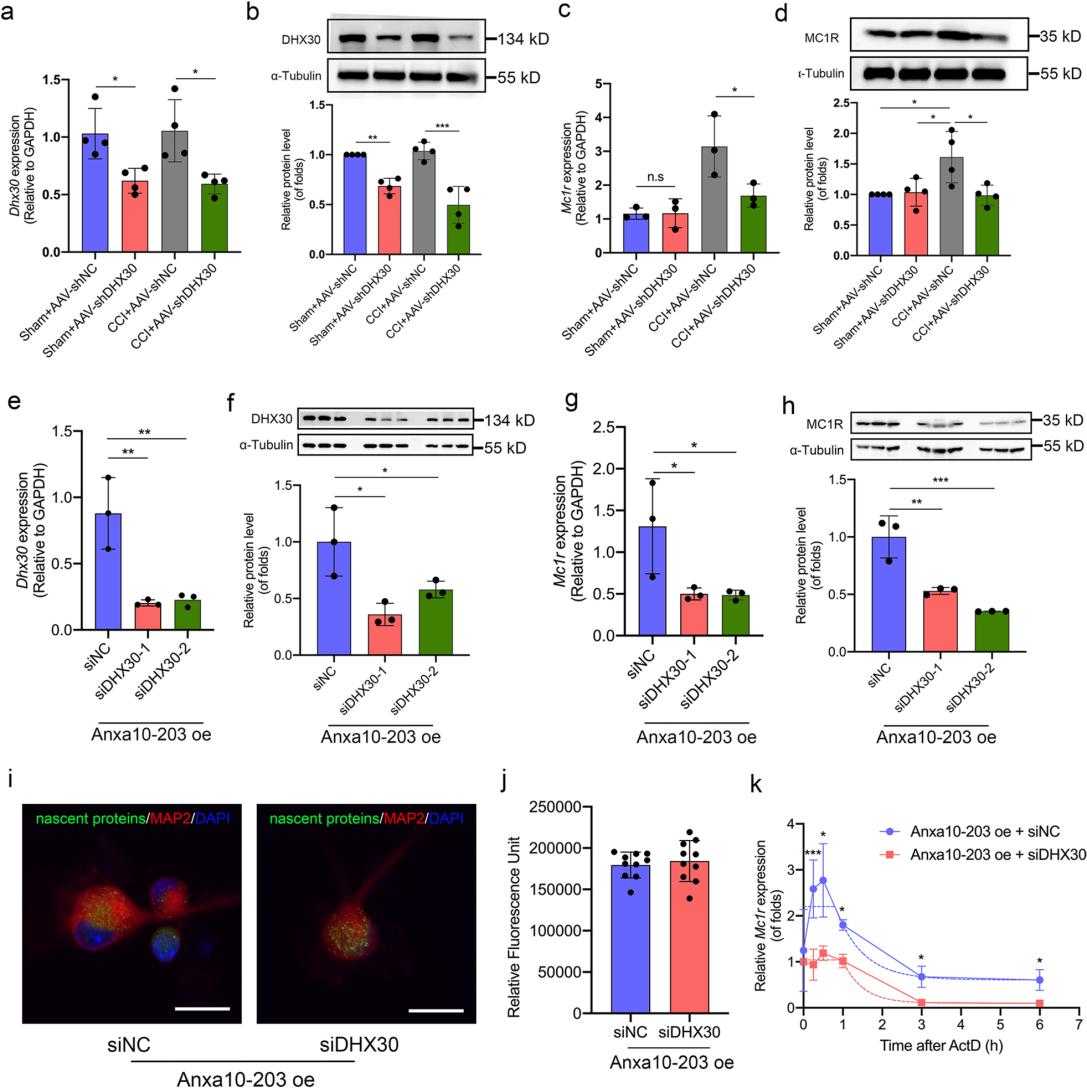
Anxa10-203 over-expression enhanced *Mc1r* mRNA stability through DHX30. **a**, **b** RNA and protein expression of DHX30 in Sham and CCI mice with or without DHX30 silenced. *P < 0.05, **P < 0.01, ***P<0.001, *n* = 4/group. **c**, **d** Expression of Mc1r in Sham and CCI-ION mice with or without DHX30 silenced was assessed by RT-qPCR (**c**) and WB (**d**). *P < 0.05, **P < 0.01, *n* = 3/group for RT-qPCR, *n*=4/group for WB. **e**, **f** The expression of DHX30 mRNA (**e**) and protein (**f**) were evaluated in the TGNs with DHX30 knockdown and Anxa10-203 over-expression *in vitro*. *P < 0.05, **P < 0.01, *n* = 4/group. **g**, **h** The expression of Mc1r mRNA (**g**) and protein (**h**) was evaluated in TGNs with DHX30 silenced and Anxa10-203 over-expressed *in vitro*. *P < 0.05, **P < 0.01, ***P < 0.001, *n* = 4/group for RT-qPCR, *n*=3/group for WB. **i** Representative images of nascent protein in TGNs of the two groups. Scare bar = 20 μm. **j** Quantitative analysis of fluorescence intensity of nascent protein in the two groups. *n* = 10/group. **k** The decay of Mc1r mRNA in different groups. *P < 0.05, ***P < 0.001, *n* = 4/group. oe: over-expression, n.s: no significance

To explore the function of DHX30 recruited by Anxa10-203 in Anxa10-203/DHX30/*Mc1r* complex. We first knocked down DHX30 in the TG and verified the down-regulated DHX30 mRNA and protein in Fig. 6a-b. Then MC1R expression was assessed, and the results showed that compared with the Sham+AAV-shNC group, the MC1R mRNA and protein remained unchanged in the Sham+AAV-shDHX30 group, while DHX30 knockdown in the CCI-ION mice led to a decrease in MC1R expression (Fig. 6c-d). Therefore, DHX30 knockdown induced the decrease of MC1R expression under the condition of Anxa10-203 over-expression. To explore how DHX30 knockdown with Anxa10-203 over-expression induced the decrease in MC1R expression, we over-expressed Anxa10-203 and knocked down DHX30 in the TGNs to simulate the condition *in vivo* (Fig. 6e-f). Consistent with the results *in vivo*, DHX30 silencing caused a decrease in MC1R mRNA and protein expression (Fig. 6g-h). According to the cytoplasmic localization of DHX30 and Gene Ontology analysis in Fig. 3b, we speculated that DHX30 played a role in post-transcriptional processes. The effects of DHX30 on mRNA translation were evaluated by a nascent protein synthesis assay. Fluorescence images showed that nascent proteins (green dots) were in the cytoplasm of TGNs transfected with siNC or siDHX30 under the condition of Anxa10-203 over-expression (Fig. 6i). Then the green fluorescence intensity was quantitatively analyzed, and no significant difference was observed in the two groups, which indicated that DHX30 recruited by Anxa10-203 did not affect mRNA translation (Fig. 6j). At the transcription level, we blocked nascent mRNA synthesis using ActD, and the decay of *Mc1r* mRNA was detected by RT-qPCR and analyzed by nonlinear regression analysis. A lower level of *Mc1r* in the Anxa10-203 oe + siDHX30 group was observed at all time points compared with the Anxa10-203 oe + siNC group, and the half-life of *Mc1r* mRNA in the Anxa10-203 oe + siDHX30 group is shorter (t_1/2_ (Anxa10-203 oe + siNC) = 0.49, *R*^2^ =0.53; t_1/2_ (Anxa10-203 oe + siDHX30) = 0.35, *R*^2^ =0.89, Fig. 6k).

## Discussion

In this study, we focused on the function of lncRNAs in the TG of mice with orofacial pain. CCI and nerve transection provide stable and repeatable models for preclinical studies of NP [32, 33]. Considering entrapment, compression, and stretching are common causes of NP in the clinic, CCI-ION is an appropriate model for orofacial NP research [34]. Based on the preclinical models, our study confirmed that Anxa10-203 was up-regulated in the TG of CCI-ION mice and the knockdown of Anxa10-203 relieved pain, suggesting that Anxa10-203 in the TG played a vital role in orofacial NP. Further investigation indicated that Anxa10-203 interacted with DHX30 and enhanced *Mc1r* mRNA stability, resulting in the up-regulated expression of MC1R that promoted the TGNs excitability and orofacial NP. These findings provided primary data for novel potential targets of NP intervention.

As a molecular scaffold, lncRNAs interact with macro-molecules and form functional complexes, so it is easy to speculate the interaction between lncRNAs and RNA helicase [13, 16]. Our study confirmed the interaction between DHX30 and lncRNA Anxa10-203 and revealed its function in *Mc1r* mRNA decay. We further verified the Anxa10-203/DHX30 complex and preliminarily showed that Exon 8 of Anxa10-203 may be the main sequence interacting with DHX30 using RNA pull-down. However, the RNA synthesized *in vitro* and the RNA *in vivo* may be different in secondary and tertiary structures, and most RNA helicases lack inherent sequence or structural specificity [35, 36]. Therefore, a multidisciplinary method was needed to characterize the exact binding site of DHX30 and Anxa10-203. The combination of X-ray crystallography and high-resolution cryo-electron microscopy should be an appropriate strategy [37].

DHX30 belongs to RNA helicases, which are generally believed to unwind secondary, tertiary structures of RNA, and participate in RNA-RNA, and RNA-protein interaction [38]. The role of DHX30 in the metabolism of RNA and protein has been reported in different cells [39]. In this study, we explored the role of DHX30 in post-transcriptional regulation in TGNs. It was found that the half-life of *Mc1r* mRNA decreased after DHX30 knockdown with Anxa10-203 over-expression, but no obvious impact on the synthesis of nascent proteins was observed. Therefore, DHX30 was involved in maintaining the stability of *Mc1r* mRNA under the experimental conditions. However, previous studies have found that over-expression of DHX30 mutants in HEK293T and human U2OS cells reduced global translation [14, 40]. The same result was also confirmed in cancer cell line models of HCT116, SJSA1, and MCF7 [41, 42]. As observed in other helicase families, the interaction between DDX3 and eIF4G acted as both inhibitor and activator of translation [43]. Thus the ultimate function of DHX30 may depend on the interaction between different RNA-binding proteins or complexes [38]. In addition, although RNA helicase had some cross-species functions, it was worth noting that these experimental data came from different species, cells, and conditions, therefore, the contradictory results are not unexplainable.

Like most G protein-coupled receptors (GPCRs), MC1R is cascaded and coupled with several intracellular signals [44]. It was reported that activation of MC1R led to increased cAMP expression in CATH.a cell line and rat primary hypothalamic neurons [20, 45]. The increase of cAMP in neurons is usually related to increased pain, and drugs that reduce cAMP synthesis have an analgesic effect [46, 47]. Based on the results of the above studies, MC1R seems to promote neuropathic pain by activating cAMP, but there is no direct evidence that MC1R-induced cAMP is involved in the occurrence of neuropathic pain, and the derivation based on the conclusions of different studies needs to be cautious. In addition, some studies suggested that MC1R-mediated cAMP signal cascade and the transcription of Mc1r mRNA, each appeared to primarily affect hair color and pain sensitivity respectively [23]. Therefore, whether MC1R-induced cAMP/AKT pathway activation is involved in neuropathic pain is an interesting question, and complete research is needed to explore this issue.

Recently, several studies showed that MC1R was located in the DRG, dorsal horn, periaqueductal gray matter (PAG), hypothalamus, dorsal hippocampus, and cerebral cortex, among which DRG, dorsal horn, and PAG are important nociceptive relays [48, 49]. These results indicated the physiological basis of the involvement of MC1R in pain. Mechanically, it was reported that MC1R participated in nociceptive sensory by affecting melanocortin/opioid signaling balance [20]. α-MSH, the main endogenous agonist of MC1R, is produced by the decomposition of pro-opiomelanocortin (POMC), while POMC decomposition also yields β-endorphin, an endogenous opioid agonist [23, 50]. Thus, it is plausible that reduced levels of MC1R may signal for an upregulation of POMC, which increases the availability of the opioid agonist and its analgesic activity [23]. However, whether this mechanism is also applicable in the peripheral nervous system needs further research, because the source of endogenous POMC in the peripheral nervous system is not clear [51]. This study confirmed that MC1R activation enhanced the intrinsic excitability of neurons in the peripheral ganglion and contributed to nociceptive sensory. Since the pigment function of MC1R seems less important in the TG, MC1R may be a specific target for pain intervention with little effect on physiological function.

## Conclusions

This study demonstrated that Anxa10-203 was a significantly up-regulated lncRNA in the TG of mice with orofacial NP. The up-regulated Anxa10-203 was associated with MC1R expression. Mechanistically, Anxa10-203 recruited DHX30 to maintain the stability of *Mc1r*, resulting in the upregulation of MC1R, which contributed to the enhancement of TGNs activity and orofacial NP. These findings enriched our understanding of the function of LncRNA Anxa10-203 and MC1R in nociception and provided evidence for potential targets for NP intervention.

### Supplementary Information

**Supplementary Material 1.** 

## Data Availability

The data supporting the findings of this study are available from the corresponding author upon reasonable request.

## Abbreviations

LncRNAs: Long non-coding RNAs
TG: Trigeminal ganglion
TGNs: Trigeminal ganglion neurons
DHX30: DExH-box helicase 30
CCI-ION: Chronic constriction injury of the infraorbital nerve
ION: Infraorbital nerve
NP: Neuronopathic pain
MC1R: Melanocortin receptor 1
HWT: head withdrawal threshold
AP: Action potential
LSCM: Laser scanning confocal microscope
MS: Mass spectrometry
BMS: BMS-470539
AHP: Afterhyperpolarization
