# Supplementary material for: LncRNA Anxa10-203 enhances Mc1r mRNA stability to promote neuropathic pain by recruiting DHX30 in the trigeminal ganglion
Source: J Headache Pain. 2024 Mar 4;25(1):28. doi: 10.1186/s10194-024-01733-2 (PMC10910797; doi:10.1186/s10194-024-01733-2)
Supplement: Supplementary file 1 — Supplementary Material 1. [file 10194_2024_1733_MOESM1_ESM.docx]

**Additional file 1**

Supplementary Figures

**
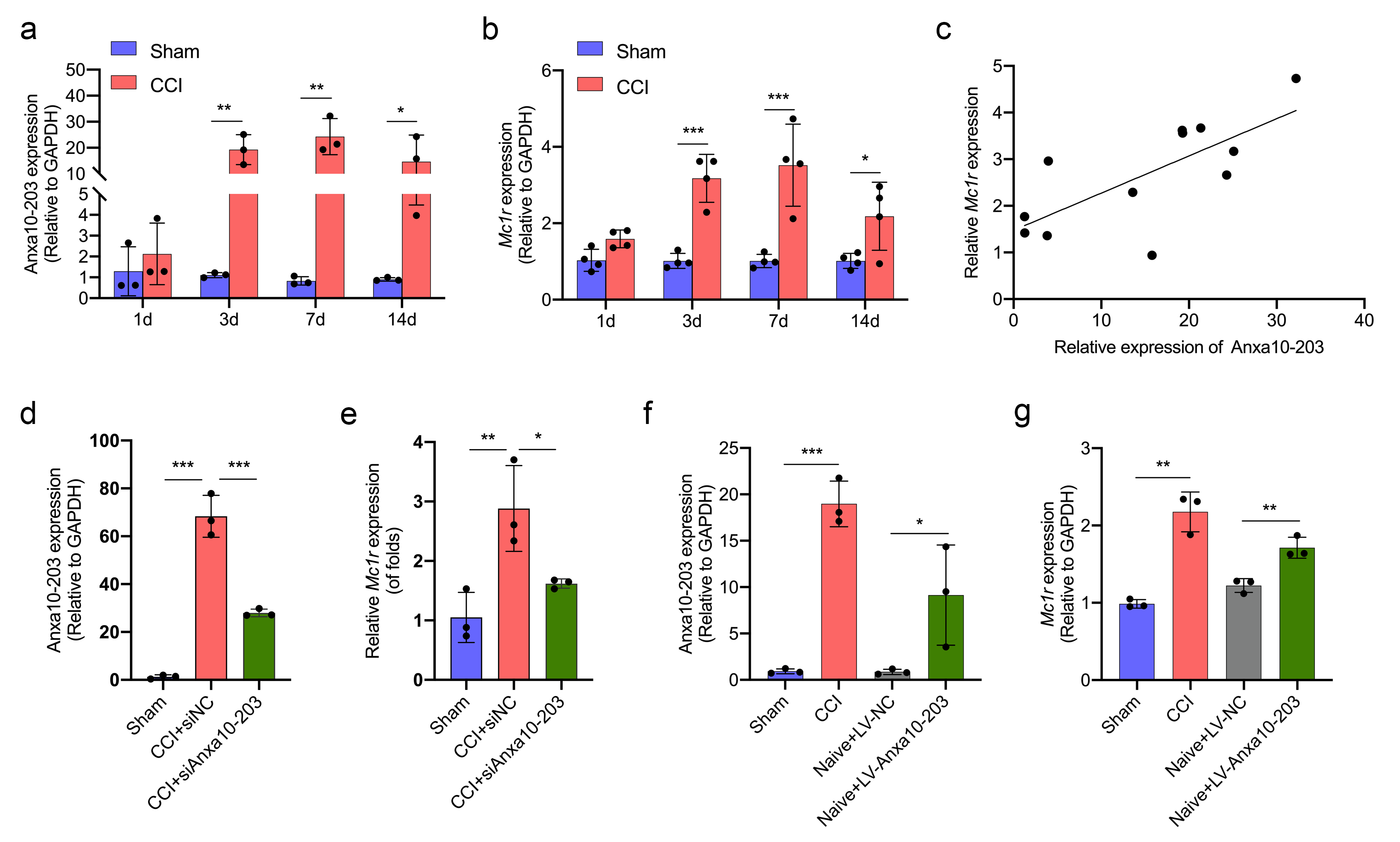
**

Supplementary Figure 1. Anxa10-203 expression was associated with MC1R in the TG of female mice. (a) The expression of Anxa10-203 in the TG of female mice at days 1, 3, 7, and 14 after CCI-ION. *P < 0.05, **P < 0.01, n = 3/group. (b) The expression of Mc1r in the TG of female mice at days 1, 3, 7, and 14 after CCI-ION. *P < 0.05, ***P < 0.001, n = 4/group. (c) Correlation analysis between Anxa10-203 and Mc1r in the TG of female mice. R2 = 0.521, P = 0.008, n = 12/group. (d) The konckdown of Anxa10-203 after siAnxa10-203 injection was verified. ***P < 0.001, n = 3/group. (e) The Anxa10-203 knockdown induced decreased Mc1r. *P < 0.05, **P < 0.01, n = 3/group. (f) The over-expression of Anxa10-203 after LV-Anxa10-203 injection was verified. *P < 0.05, ***P < 0.001, n = 3/group. (g) The Anxa10-203 over-expression induced increased Mc1r in female mice. **P < 0.01, n = 3/group.


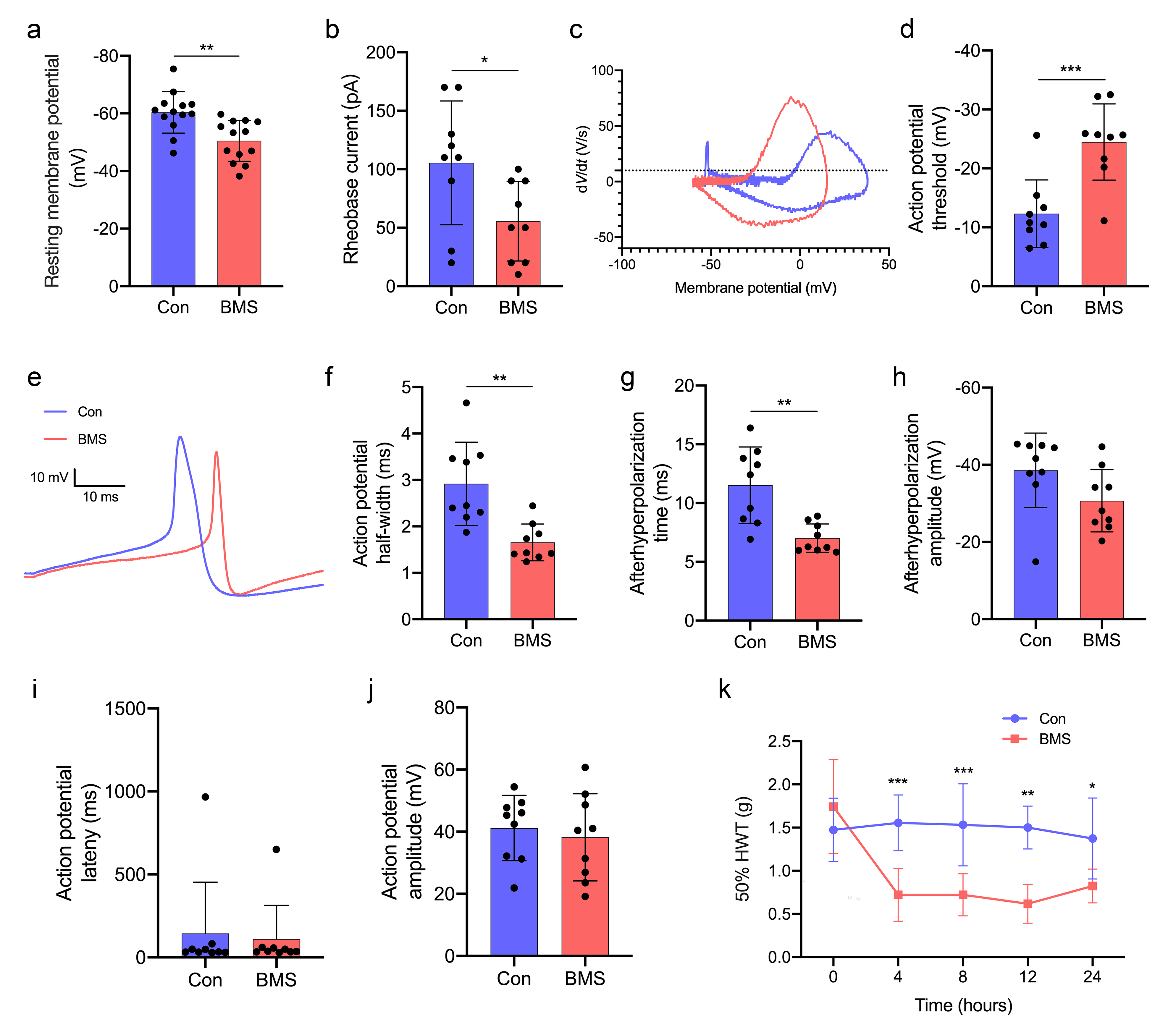


Supplementary Figure 2. MC1R activation promoted the intrinsic excitability of TGNs and orofacial NP. (a) The RMP of the Con and BMS (100 nM) groups. ***P* < 0.01, n = 13/group. (b) The rheobase current of different groups. ****P*<0.001, n = 9/group. (c) Representative phase plane plots in the Con and the BMS group. (d) The AP threshold in different groups. ****P*<0.001, n=9/group. (e) Representative AP traces evoked by rheobase currents in the two groups. (f-j) Data of AP half-width (f), AHP amplitude (g), AHP time (h), AP latency (i), and AP amplitude (j). **P* < 0.05, ***P* < 0.01, n = 9/group. (k) MC1R activation decreased HWT in mice. **P* < 0.05, ***P* < 0.01, ****P* < 0.001, n = 7/group.
